# The impact of antimicrobial use regulations on antimicrobial resistance among *Salmonella* isolates from bovine samples submitted to a veterinary diagnostic laboratory in Central New York

**DOI:** 10.1016/j.onehlt.2025.101087

**Published:** 2025-06-02

**Authors:** Maya Craig, Kevin J. Cummings, Claudia Cobo-Angel, Casey L. Cazer, Melissa S. Aprea, Rebecca J. Franklin-Guild

**Affiliations:** a Department of Public and Ecosystem Health, College of Veterinary Medicine, Cornell University, Ithaca, NY, United States of America; b Department of Clinical Sciences, College of Veterinary Medicine, Cornell University, Ithaca, NY, United States of America; c International Centre for Antimicrobial Resistance Solutions (ICARS), Copenhagen, Denmark; d Animal Health Diagnostic Center, Cornell University, Ithaca, NY, United States of America

**Keywords:** *Salmonella enterica*, Antimicrobial use, Antimicrobial resistance, Public health, Dairy cattle

## Abstract

In recognition that antimicrobial resistance in human pathogens may stem from antimicrobial use in agricultural settings, the United States Food and Drug Administration (FDA) ordered restrictions on antimicrobial usage (AMU) in food-producing animals. In 2012 the FDA restricted the extra-label use of third-generation cephalosporins, and in 2017 the FDA mandated veterinary oversight for the use of antimicrobials in the feed and water of food-producing animals and eliminated production-related uses. However, the impact of these restrictions on the antimicrobial resistance status of important pathogens, such as *Salmonella*, remains unclear. To address this gap in knowledge, we analyzed veterinary diagnostic laboratory data on 2413 *Salmonella* isolates from submitted bovine samples. We fitted logistic regression models to evaluate changes in proportions of antimicrobial-resistant isolates, and we used accelerated failure time (AFT) models to determine changes in minimum inhibitory concentration (MIC) values. Our analysis revealed the 2012 AMU restriction to be associated with a decrease in the odds of resistance to chlortetracycline (OR = 0.49; 95 % CI = 0.28–0.86), oxytetracycline (OR = 0.47; 95 % CI = 0.27–0.82), and neomycin (OR = 0.45; 95 % CI = 0.25–0.80). Furthermore, we found significant decreases in MIC values for chlortetracycline (CR = 0.74; 95 % CI = 0.62–0.87) and oxytetracycline (CR = 0.64; 95 % CI = 0.56–0.73) for the same AMU restriction. We found a significant association between the 2017 AMU restriction and decreased odds of resistance to florfenicol (OR = 0.28; 95 % CI = 0.09–0.92). *Salmonella* serotype was an important predictor of resistance to all antimicrobials assessed via logistic regression or AFT models. Overall, our study suggests that in the region served by the laboratory, AMU restrictions have either had no detectable effect or are associated with decreasing AMR and MIC trends for *Salmonella* isolated from bovine samples, depending on the antimicrobial.

## 1 Introduction

Antimicrobial resistance (AMR) is a major One Health concern, and mitigating its emergence and spread is crucial for public health. The overuse and misuse of antimicrobials has been shown to lead to an increase in antimicrobial-resistant organisms in all areas of health [1,2] However, the usage of antimicrobials in agriculture is particularly controversial because of the ongoing debate that it is a main driver of increasing AMR [3]. Some researchers have argued that there is no clear linkage between antimicrobial usage (AMU) in agricultural settings and drug-resistant infections in humans, while others argue that there is a connection between the two [4]. Resistant bacteria in the gut flora of livestock, either resistant pathogens or commensal organisms carrying mobile resistance genes, can spread into the environment and to the human population via food production pathways or direct contact [5,6].

*Salmonella enterica* is frequently isolated from agricultural settings, including dairy cattle populations, and has often demonstrated antimicrobial resistance [7]. Although more than 2600 *Salmonella* serotypes have been identified, there are only a few that cause most cases of disease in dairy cattle and humans [8]. For example, *Salmonella* Typhimurium and *Salmonella* Newport are two serotypes that are linked to clinical disease in both human and dairy cattle populations [2,9,10]. *Salmonella* infections in humans typically cause gastroenteritis symptoms that resolve without medical intervention [8]. However, some *Salmonella* infections may be severe and require hospitalization [11,12]. Because of the overlap of *Salmonella* serotypes found on dairy farms and isolated from human patients with laboratory-confirmed salmonellosis, it is important to maintain proper and effective disease outbreak management on farms while preserving responsible antimicrobial stewardship [13,14].

In 2012, the U.S. Food and Drug Administration (FDA) implemented restrictions on the use of cephalosporins in agriculture, allowing use to treat or control an extra-label disease indication only when this use adheres to the labeled dose, route, and duration [15]. Maintaining the effectiveness of cephalosporins is of particular concern because agents belonging to this drug class are identified by the FDA and World Health Organization as medically important antimicrobials for human health and are first-line drugs for treating invasive salmonellosis in people [16,17]. Reducing the use of cephalosporins in agriculture can lessen the likelihood of cross-resistance between cephalosporins that are used in animals and molecularly similar cephalosporins that are used in humans [18]. In 2017, another restriction on antimicrobial use in agriculture went into effect as a part of the Veterinary Feed Directive (VFD). This restriction required veterinary oversight for all medically important antimicrobials used in the feed or water of food-producing animals, including dairy cattle, and it eliminated use for production reasons such as growth promotion [19].

In an effort to understand how AMR trends have changed in response to antimicrobial use, studies have used logistic and linear regression methods to assess changes in AMR trends and whether these changes can be predicted by covariates [20,21]. For logistic regression modeling, studies use susceptibility status (resistant or susceptible) as a binary outcome and include at least one predictor variable that indicates the start of FDA restrictions [21]. Logistic regression, like other multivariable modeling approaches, is beneficial in that it can control for confounders while quantifying the impact of predictors of interest. However, the dichotomization of data may result in the loss of information and statistical power, since it turns a continuous variable (MIC) into a binary variable (susceptible vs resistant) and it is thus important to perform additional analyses to expand our understanding of the impact of FDA regulations [22]. Linear regression can reduce the impact of the loss of information due to dichotomization because it uses a continuous variable rather than a binary variable as an outcome [23]. Many researchers have opted to use linear regression to analyze MICs as a means of understanding associations between FDA restrictions and AMR trends [20]. However, when using veterinary diagnostic laboratory data, there is an inherent lack of data due to the two-fold dilution method in determining MIC values. For example, to determine MIC values, antimicrobial concentrations are doubled in a stepwise manner until there is no more observed bacterial growth. Therefore, the true MIC value can lie anywhere between the recorded MIC value and the next lower concentration tested. Thus, it is important to use statistical models that can handle these gaps in data.

One way to address the gaps in MIC data is to treat the observations as censored data and utilize Cox proportional hazards or accelerated failure time (AFT) models for a survival model type analysis. Cobo et al. used parametric survival models to explore MIC trends among *Escherichia coli* isolated from cats during a 14-year period [24]. Osman et al. used a semi-parametric Cox proportional hazards model to analyze the MIC values of *Escherichia coli* isolated from canine samples during a longitudinal study [25]. Both studies used data from veterinary diagnostic laboratories for their respective analyses. These approaches allow an evaluation of MIC values despite the gaps in information due to two-fold serial dilutions.

Here, we used logistic regression and AFT models to examine AMR trends in *Salmonella* isolates from dairy cattle in response to federal AMU restrictions, based on veterinary diagnostic laboratory data. The findings of this study will help to mend the gap in knowledge about the effectiveness of federal restrictions on reducing AMR in livestock.

## 2 Materials and methods

### 2.1 Study design

Data were collected retrospectively for all bovine *Salmonella* isolates from samples submitted to the Cornell University Animal Health Diagnostic Center (AHDC) between January 1, 2007, and December 31, 2022. The dataset included information that was described in a previous study, with the addition of samples submitted to the AHDC in 2022 [26]. Briefly, the complete dataset consisted of sample accession numbers, dates of sample submission and *Salmonella* isolation, sample type, U.S. state of sample origin, serotype, antimicrobial MIC values, and the interpretations of the MIC values (susceptible, intermediate, or resistant). For the purposes of this analysis, isolates for which antimicrobial susceptibility testing was not performed were removed from the dataset.

### 2.2 Microbiologic procedure for *Salmonella* detection

*Salmonella* isolation and antimicrobial susceptibility testing were completed by the AHDC. The methods were previously described in Craig et al. [26]. Briefly, enteric samples were plated to Levine eosin methylene blue (EMB) agar and Brilliant Green (BG) agar and struck for isolation. Approximately 1 g of the sample, or the entire submitted swab, was also inoculated into 10 mL of tetrathionate (TT) broth (2007–2013) or 10 mL of Rappaport–Vassiliadis Soya (RVS) (2013−2022) broth for enrichment purposes. The enrichment broth was incubated overnight at 40–44 °C and sub-cultured the following day to Brilliant Green with novobiocin (BGN) (2007–2013) or BG (2013–2022) and Xylose Lysine Tergitol 4 (XTL-4) (2007–2013) or Xylose Lysine Deoxycholate (XLD) (2013–2022) agar plates. All culture plates were incubated overnight at 33–37 °C and evaluated at 18–24 h. Suspicious colonies were tested to confirm or rule out *Salmonella* species by the Sensititre Automated Identification System (Thermo Fisher Scientific; Waltham, MA) (2007–2012) or MALDI-TOF mass spectrometry (MS) using the Bruker MALDI Biotyper® System (Bruker Scientific, Billerica, MA) (2013–2022), along with conventional biochemical screens as necessary for unusual isolates. To serogroup the isolates, single-colony streaking was used, and Statens Serum Institute antisera were used for slide agglutination. Tryptic Soy agar (TSA) slants were also prepared to submit the isolates for serotyping at a referral laboratory (National Veterinary Services Laboratories [NVSL, United States Department of Agriculture, Ames, IA]).

Non-enteric samples received by the laboratory for *Salmonella* culture were plated to Tryptic Soy with 5 % sheep blood (BAP) agar, chocolate agar, Levine EMB agar, and Columbia CNA agar and struck for isolation. Culture plates were incubated overnight at 33–37 °C and evaluated at 18–24 h and 2 days following plating. Suspicious colonies were tested to confirm or rule out *Salmonella* species by the Sensititre Automated Identification System (Thermo Fisher Scientific; Waltham, MA) (2007–2012) or MALDI-TOF MS using the Bruker MALDI Biotyper® System (Bruker Scientific) (2013–2022), along with conventional biochemical screens as necessary for unusual isolates.

### 2.3 Antimicrobial susceptibility testing

Antimicrobial susceptibility of confirmed *Salmonella* isolates was determined using the broth microdilution method. Minimum inhibitory concentrations (MIC) were established for each isolate against the following antimicrobial agents: ampicillin, ceftiofur, chlortetracycline, enrofloxacin, florfenicol, gentamicin, neomycin, oxytetracycline, spectinomycin, sulfadimethoxine, and trimethoprim/sulfamethoxazole; all other antimicrobials were excluded from this analysis due to missing values. MIC values for ampicillin, ceftiofur, gentamicin, spectinomycin, sulfadimethoxine, and trimethoprim/sulfamethoxazole were interpreted using the National Antimicrobial Resistance Monitoring System (NARMS) breakpoints [27]. Chlortetracycline, enrofloxacin, florfenicol, neomycin, and oxytetracycline MIC values were interpreted using CLSI breakpoints. Isolates were categorized as being resistant or susceptible to each agent; those few isolates characterized as intermediate were categorized as susceptible.

### 2.4 Statistical analysis

The software program RStudio (version 4.3.2) was used to analyze the dataset of *Salmonella* isolates. Descriptive analysis was performed to investigate the general characteristics of the dataset. Chi-squared tests were completed to assess independent associations between antimicrobial susceptibility and the implementation of AMU restriction by the FDA in 2012 and 2017. *P*-values <0.05 were considered statistically significant in all analyses.

To evaluate the impact of federal restrictions on AMR trends of *Salmonella* isolates, logistic regression models were fitted for each antimicrobial included in the study; these models assumed that federal AMU restrictions were implemented uniformly in all U.S. states. Each logistic regression model included serotype as a categorical variable (4, [5],12:i:-, Cerro, Dublin, Newport, Typhimurium, and other), year of isolation as a continuous variable, and a categorical variable to indicate the presence or absence of a federal restriction (no restriction, 2012 restriction on cephalosporin extra-label usage, and 2017 restriction on antimicrobials in feed or water) as the covariates, and susceptibility as the binary outcome (resistant or susceptible). The serotype categories were selected based upon the most frequently isolated serotypes identified in Craig et al., 2023 [26]. Montevideo was included in the ‘other’ category due to the lack of variation in resistant isolates.

The following variables were used as reference categories for all logistic regression models: AMU restriction = no restriction, year = 0, and serotype = other. Isolates that had missing values were not considered for model fitting. The antimicrobial concentration required to inhibit the growth of 50 % (MIC_50_) and 90 % (MIC_90_) of isolates tested in each time period was calculated. Numerical MIC values were recorded as a value (e.g., MIC = 4), a less than or equal to value (e.g., MIC ≤4), or a greater than value (e.g., MIC >4). For each isolate, an MIC interval was constructed. If the isolate was recorded as a value, the MIC interval was that given value as the right limit and the concentration tested prior to that given value as the left limit (e.g., MIC = 4; (2,4)). MICs were tested in 2-fold increases, so the prior concentration was always 50 % of the right limit. For isolates where the right limit was unknown (e.g., MIC >4), the MIC interval was the highest concentration tested as the left limit, and the right limit was considered right censored (e.g., (4, infinity)). For isolates where the left limit was unknown (ex: MIC ≤4), the interval was the highest concentration tested as the right limit, and the left limit was censored.

An accelerated failure time model (AFT) model approach was used to assess changes in MIC values in association with the implementation of federal restrictions on AMU, as previously described by Cobo et al. [24]. The model assumes that the covariates multiplicatively increase or decrease MIC values by some constant. A concentration ratio less than 1 indicates lower MIC values and thus decreasing resistance. A concentration ratio greater than 1 indicates higher MIC values and thus increasing resistance. A concentration ratio equal to 1 indicates no change in MIC values across the study period. R package icenReg (version 2.0.16) was used to fit the AFT models. Weibull, exponential, log-normal, and log-logistic models were evaluated using Akaike information criterion (AIC) values to determine the best fitting model distribution. The covariates in the model included serotype, year, and an indicator variable for the presence or absence of federal restrictions. The outcome variable was the log(MIC). If more than 85 % of isolates had the same MIC value for an antimicrobial, that antimicrobial was not considered for AFT modeling due to the lack of variability. The following variables were considered reference levels: restriction = no restriction, year = 0, and serotype = other. A sensitivity analysis for the AFT models was done to evaluate the impact of censorship on model coefficients. Right limit censored values were replaced with 2×, 5×, or 10× of the highest antimicrobial concentration tested.

## 3 Results

### 3.1 Descriptive analysis

The total number of isolates available for analysis was 2413. The number of isolates tested for antimicrobial susceptibility before any federal AMU restriction (before 2012) was 1220, the number of isolates tested in between the implementation of the 2012 and 2017 restrictions was 966, and the number of isolates tested after the implementation of both restrictions was 227.

**Fig. 1:**
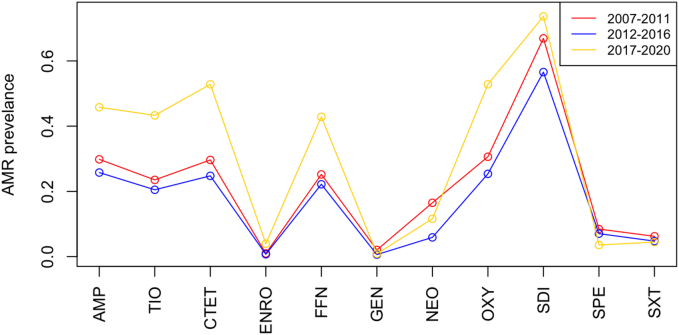
The proportion of isolates that are resistant to eleven different antimicrobials. AMP = ampicillin, TIO = ceftiofur, CTET = chlortetracycline, ENRO = enrofloxacin, FFN = florfenicol, GEN = gentamicin, OXY = oxytetracycline, SDI = sulfadimethoxine, SPE = spectinomycin, SXT = trimethoprim/sulfamethoxazole.

The proportion of isolates resistant to the following antimicrobials was significantly different between at least two of the time periods: ampicillin (*p* < 0.0001), ceftiofur (p < 0.0001), chlortetracycline (p < 0.0001), enrofloxacin (*p* = 0.0002), florfenicol (p < 0.0001), gentamicin (p < 0.0001), neomycin (p < 0.0001), oxytetracycline (p < 0.0001), sulfadimethoxine (*p* < 0.001), and spectinomycin (*p* = 0.03). There was no significant difference in the proportion of isolates resistant to trimethoprim/sulfamethoxazole (*p* = 0.2) across each of the time periods (Fig. 1).

The MIC_50_ was higher in the 2017–2022 time period in comparison to the 2007–2011 period for ampicillin, chlortetracycline, florfenicol, oxytetracycline, and sulfadimethoxine. The MIC_90_ was lower in 2012–2016 for neomycin in comparison to the 2007–2011 period. The MIC_90_ was lower in 2012–2016 for spectinomycin in comparison to the 2017–2022 period. There were no observed changes in the MIC_50_ or MIC_90_ for the remainder of other antimicrobials that were included in the study (Table 1, Table 2).

**Table 1:** The required MIC to inhibit for 50 % or 90 % of *Salmonella enterica* isolated from bovine samples submitted to the AHDC during different time periods.

	2007–2011		2012–2016		2017–2022	
	MIC_50_	MIC_90_	MIC_50_	MIC_90_	MIC_50_	MIC_90_
Ampicillin	1	>16	1	>16	2	>16
Ceftiofur	1	>8	1	>8	1	>8
Chlortetracycline	1	>8	1	>8	>8	>8
Enrofloxacin	0.12	0.12	0.12	0.12	0.12	0.12
Florfenicol	2	>8	2	>8	4	>8
Gentamicin	1	1	1	1	1	1
Neomycin	4	>32	4	4	4	4
Oxytetracycline	1	>8	1	>8	>8	>8
Spectinomycin	32	64	32	64	32	32
Sulfadimethoxine	256	>256	>256	>256	>256	>256
Trimethoprim/Sulfamethoxazole	2	2	2	2	2	2

**Table 2:**
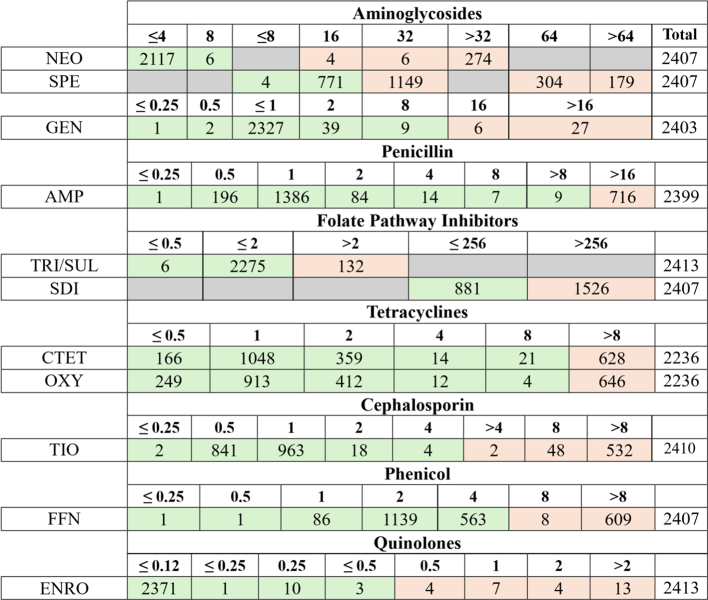
Number of isolates by MIC values for eleven antimicrobials stratified by drug class.

**Key:** green = susceptible, red = resistant. Gray = not tested.

Abbreviations: AMP = ampicillin, TIO = ceftiofur, CTET = chlortetracycline, ENRO = enrofloxacin, FFN = florfenicol, GEN = gentamicin, OXY = oxytetracycline, SDI = sulfadimethoxine, SPE = spectinomycin, SXT = trimethoprim/sulfamethoxazole.

### 3.2 Logistic regression models

More than 95 % of isolates that were tested for susceptibility against gentamicin and enrofloxacin were susceptible to these antimicrobials (Table 2). Therefore, resistance to these antimicrobials was not evaluated by logistic regression modeling. When assessing resistance to sulfadimethoxine, *Salmonella* Dublin isolates were removed because all Dublin isolates were resistant to this antimicrobial, which caused model convergence issues **(**Table 3**).** *Salmonella* Newport susceptibility data for ceftiofur were removed from the dataset because more than 60 % of Newport isolates were censored (e.g., had an MIC value equal to or less than the smallest concentration tested) and therefore lacked sufficient variation for the logistic regression model and subsequent AFT model (Table 4).

**Table 3:** Proportion of *Salmonella* Dublin isolated from bovine samples that are resistant to Sulfadimethoxine.

	Resistance	Susceptible
Dublin	276	0

**Table 4:** Distribution of *Salmonella* Newport ceftiofur MIC values.

	**≤ 0.25**	**0.5**	**1**	**2**	**4**	**>4**	**8**	**>8**	**Total**
TIO	0	3	9	0	4	1	0	137	154

The 2012 restriction of extra-label cephalosporin use was significantly associated with a decrease in odds of resistance to chlortetracycline, neomycin, and oxytetracycline, relative to the 2007–2011 baseline (Table 5). The 2017 restriction of antimicrobials in the feed or water of livestock was significantly associated with a decrease in odds of resistance to florfenicol, in comparison to the 2007–2011 baseline (Table 5).

**Table 5:** Logistic regression models evaluating the impact of federal restrictions on AMU on *Salmonella enterica* isolated from bovine samples submitted to the AHDC.

	Odds Ratio (OR)	95% CI	P-value
**Ampicillin**			
2012 restriction	0.73	(0.43, 1.24)	0.25
2017 restriction	0.49	(0.17, 1.46)	0.20
Year	0.98	(0.89, 1.07)	0.60
4,[5],12:i:-	5.59	(3.73, 8.49)	**< 0.01**
Cerro	0.03	(0.02, 0.06)	**< 0.01**
Dublin	75.15	(42.78, 142.10)	**< 0.01**
Newport	29.04	(16.30, 56.63)	**< 0.01**
Typhimurium	0.46	(0.32, 0.66)	**<0.01**
**Ceftiofur**⁎			
2012 restriction	1.14	(0.52, 1.71)	0.66
2017 restriction	1.19	(0.31, 3.50)	0.78
Year	0.94	(0.84, 1.04)	0.28
4,[5],12:i:-	1.66	(1.09, 2.70)	**0.03**
Cerro	0.03	(0.01, 0.06)	**< 0.01**
Dublin	67.78	(44.70, 130.50)	**< 0.01**
Typhimurium	0.40	(0.25, 0.62)	**< 0.01**
**Chlortetracycline**			
2012 restriction	0.49	(0.28, 0.86)	**0.01**
2017 restriction	0.70	(0.17, 2.72)	0.61
Year	1.02	(0.92, 1.13)	0.66
4,[5],12:i:-	5.79	(3.80, 8.94)	**< 0.01**
Cerro	0.04	(0.02, 0.07)	**< 0.01**
Dublin	176.55	(72.17, 585.36)	**< 0.01**
Newport	30.26	(16.65, 60.76)	**< 0.01**
Typhimurium	0.28	(0.17, 0.42)	**< 0.01**
**Florfenicol**			
2012 restriction	0.62	(0.62, 2.38)	0.10
2017 restriction	0.28	(0.09, 0.92)	**0.04**
Year	1.02	(0.84, 1.06)	0.66
4,[5],12:i:-	1.87	(1.18, 3.17)	**< 0.01**
Cerro	0.03	(0.01, 0.06)	**< 0.01**
Dublin	99.01	(55.10, 192.61)	**< 0.01**
Newport	37.78	(18.32, 59.37)	**< 0.01**
Typhimurium	0.33	(0.21, 0.51)	**< 0.01**
**Neomycin**			
2012 restriction	0.45	(0.25, 0.80)	**0.01**
2017 restriction	0.84	(0.28, 2.54)	0.76
Year	0.96	(0.87, 1.06)	0.45
4,[5],12:i:-	2.47	(1.57, 3.84)	**< 0.01**
Cerro	0.01	(0.002, 0.04)	**< 0.01**
Dublin	1.09	(0.70, 1.69)	0.71
Newport	3.62	(2.46, 5.33)	**< 0.01**
Typhimurium	0.23	(0.12, 0.39)	**< 0.01**
**Oxytetracycline**			
2012 restriction	0.47	(0.27, 0.82)	**0.02**
2017 restriction	0.27	(0.15, 2.28)	0.47
Year	1.03	(0.93, 1.14)	0.53
4,[5],12:i:-	5.22	(3.44, 8.05)	**< 0.01**
Cerro	0.05	(0.03, 0.08)	**< 0.01**
Dublin	159.76	(65.40, 529.23)	**< 0.01**
Newport	27.34	(15.06, 54.85)	**< 0.01**
Typhimurium	0.26	(0.17, 0.39)	**< 0.01**
**Spectinomycin**			
2012 restriction	1.12	(0.58, 2.16)	0.74
2017 restriction	0.63	(0.15, 2.53)	0.51
Year	0.97	(0.87, 1.13)	0.56
4,[5],12:i:-	1.51	(0.93, 2.40)	0.08
Cerro	0.04	(0.01, 0.08)	**< 0.01**
Dublin	0.09	(0.03, 0.22)	**< 0.01**
Newport	0.14	(0.04, 0.33)	**< 0.01**
Typhimurium	0.37	(0.22, 0.59)	**< 0.01**
**Trimethoprim/Sulfamethoxazole**			
2012 restriction	1.43	(0.68, 3.06)	0.35
2017 restriction	2.32	(0.50, 10.91)	0.28
Year	0.87	(0.76, 1.00)	**0.05**
4,[5],12:i:-	2.61	(1.57, 4.23)	**0.01**
Cerro	0.05	(0.02, 0.12)	**< 0.01**
Dublin	0.43	(0.21, 0.83)	**0.02**
Newport	0.16	(0.04, 0.43)	**< 0.01**
Typhimurium	0.18	(0.08, 0.37)	**< 0.01**
**Sulfadimethoxine**⁎⁎			
2012 restriction	1.17	(0.78, 1.75)	0.46
2017 restriction	2.02	(0.86, 4.73)	0.11
Year	0.87	(0.80, 0.94)	**< 0.01**
4,[5],12:i:-	5.68	(3.18, 11.08)	**< 0.01**
Cerro	0.25	(0.20, 0.30)	**< 0.01**
Newport	67.42	(14.95, 1190.54)	**< 0.01**
Typhimurium	2.29	(1.66, 3.19)	**< 0.01**

⁎ *Salmonella* Newport isolates were removed from dataset for logistic regression model.

⁎⁎ *Salmonella* Dublin isolates were removed from dataset for logistic regression model.

Serotype was an important predictor of resistance to all antimicrobials evaluated by logistic regression. *Salmonella* Cerro was significantly associated with decreased odds of resistance to all antimicrobials in comparison to the reference category of “other” serotypes. *Salmonella Typhimurium* was significantly associated with decreased odds of resistance to all antimicrobials except sulfadimethoxine. *Salmonella* Newport was significantly associated with an increased odds of resistance to all evaluated antimicrobials. *Salmonella* 4, [5],12:i:- was significantly associated with an increased odds of resistance to all antimicrobials except spectinomycin. *Salmonella* Dublin was significantly associated with an increased odds of resistance to all antimicrobials except neomycin, spectinomycin, and trimethoprim/sulfamethoxazole. *Salmonella* Dublin was significantly associated with a decreased odds of resistance to spectinomycin. The year of isolation was significantly associated with a decreased odds of resistance to the folate pathway inhibitors. (Table 5). An interaction term between the restriction category and the year of isolation was assessed but not included due to collinearity violations.

### 3.3 AFT models

Because more than 85 % of isolates had the same MIC value for enrofloxacin, gentamicin, neomycin, and trimethoprim/sulfamethoxazole, changes in MIC were not evaluated using AFT modeling. Furthermore, the AHDC only tested two different concentrations for sulfadimethoxine, and therefore changes in MIC values were not evaluated for this antimicrobial (Table 2).

The implementation of the 2012 AMU restriction was found to be significantly associated with a decrease in chlortetracycline and oxytetracycline MIC values. The implementation of the 2017 AMU restriction was not associated with significant changes in MIC values for any of the antimicrobials that were evaluated using AFT models. The year of isolation was found to be significantly associated with an increase in oxytetracycline MIC values and a decrease in spectinomycin MIC values (Table 6).

**Table 6:** Accelerated Failure Time (AFT) models evaluating the impact of federal restrictions on AMU on *Salmonella enterica* isolated from bovine samples submitted to the AHDC.⁎

	Concentration Ratio (CR)	95 % CI	p-value
**Ampicillin**			
2012 restriction	0.98	(0.81, 1.19)	0.86
2017 restriction	0.85	(0.57, 1.28)	0.45
Year	0.99	(0.95, 1.02)	0.48
4, [5],12:i:-	11.04	(8.25, 14.76)	**< 0.01**
Cerro	0.51	(0.46, 0.57)	**< 0.01**
Dublin	86.01	(60.16, 122.98)	**< 0.01**
Newport	55.86	(35.62, 82.94)	**< 0.01**
Typhimurium	0.75	(0.65, 0.86)	**<0.01**
**Ceftiofur**⁎			
2012 restriction	1.07	(0.95, 1.20)	0.28
2017 restriction	1.16	(0.91, 1.49)	0.23
Year	0.99	(0.97, 1.02)	0.60
4, [5],12:i:-	1.05	(0.91, 1.23)	0.48
Cerro	0.56	(0.52, 0.60)	**< 0.01**
Dublin	17.21	(15.03, 19.69)	**< 0.01**
Typhimurium	0.92	(0.83, 1.00)	0.06
**Chlortetracycline**			
2012 restriction	0.74	(0.62, 0.87)	**< 0.01**
2017 restriction	0.84	(0.55, 1.26)	0.39
Year	1.03	(0.99, 1.06)	0.12
4, [5],12:i:-	4.98	(3.93, 6.32)	**< 0.01**
Cerro	0.40	(0.37, 0.45)	**< 0.01**
Dublin	40.35	(23.84, 68.28)	**< 0.01**
Newport	17.00	(12.19, 23.44)	**< 0.01**
Typhimurium	0.78	(0.69, 0.89)	**< 0.01**
**Florfenicol**			
2012 restriction	0.93	(0.84, 1.03)	0.14
2017 restriction	0.96	(0.78, 1.19)	0.71
Year	0.99	(0.97, 1.01)	0.06
4, [5],12:i:-	1.21	(1.06, 1.38)	**< 0.01**
Cerro	0.56	(0.53, 0.60)	**< 0.01**
Dublin	8.13	(6.83, 9.69)	**< 0.01**
Newport	6.08	(5.07, 7.28)	**< 0.01**
Typhimurium	1.04	(0.96, 1.12)	0.35
**Oxytetracycline**			
2012 restriction	0.64	(0.56, 0.73)	**<0.01**
2017 restriction	0.75	(0.53, 1.08)	0.12
Year	1.05	(1.02, 1.07)	**<0.01**
4, [5],12:i:-	5.00	(3.90, 6.43)	**< 0.01**
Cerro	0.36	(0.32, 0.40)	**< 0.01**
Dublin	49.68	(27.78, 88.83)	**< 0.01**
Newport	17.85	(12.50, 25.50)	**< 0.01**
Typhimurium	0.76	(0.66, 0.87)	**<0.01**
**Spectinomycin**			
2012 restriction	0.95	(0.88, 1.02)	0.14
2017 restriction	1.09	(0.95, 1.26)	0.22
Year	0.96	(0.96, 0.99)	**< 0.01**
4, [5],12:i:-	1.22	(1.12, 1.33)	**< 0.01**
Cerro	0.47	(0.45, 0.49)	**< 0.01**
Dublin	0.98	(0.92, 1.04)	0.50
Newport	0.81	(0.75, 0.87)	**< 0.01**
Typhimurium	1.13	(1.07, 1.20)	**< 0.01**

⁎ *Salmonella* Newport isolates were removed from dataset for AFT model.

As with the logistic regression models, the AFT models showed serotype to be an important indicator of differences in MIC values (Table 6). For all antimicrobial MICs evaluated by AFT models, *Salmonella* Cerro was found to be significantly associated with lower MIC values than the reference category. *Salmonella* Newport and 4, [5],12:i:- were associated with higher MIC values for all antimicrobials. *Salmonella* Dublin was found to be significantly associated with higher MIC values for all antimicrobials except for spectinomycin (Table 6). *Salmonella* Typhimurium was significantly associated with lower MIC values for ampicillin, chlortetracycline, and oxytetracycline but higher MIC values for spectinomycin (Table 6).

### 3.4 Sensitivity analysis

**Fig. 2:**
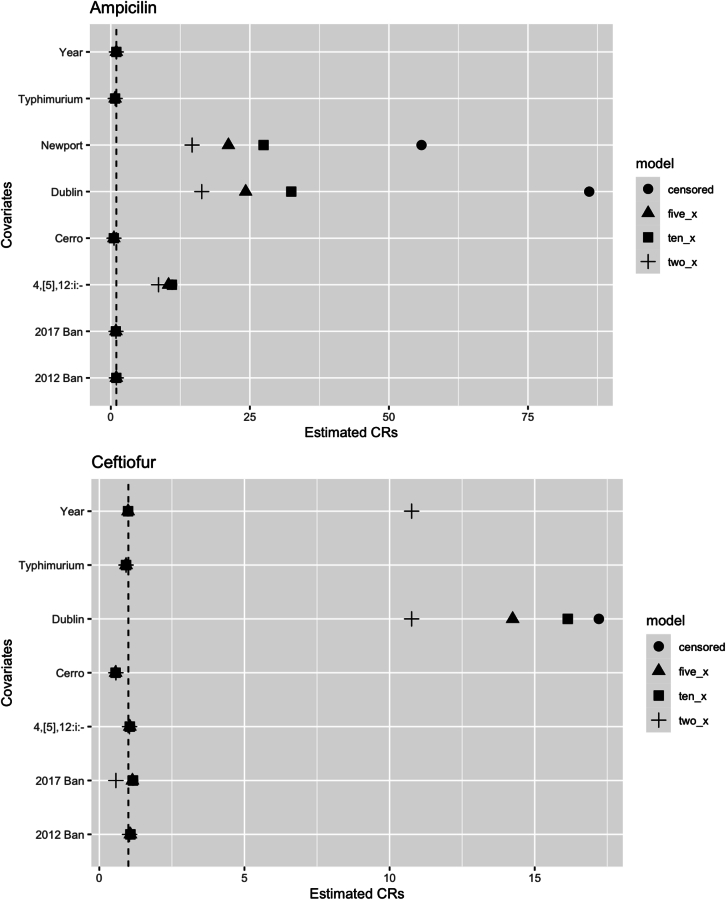

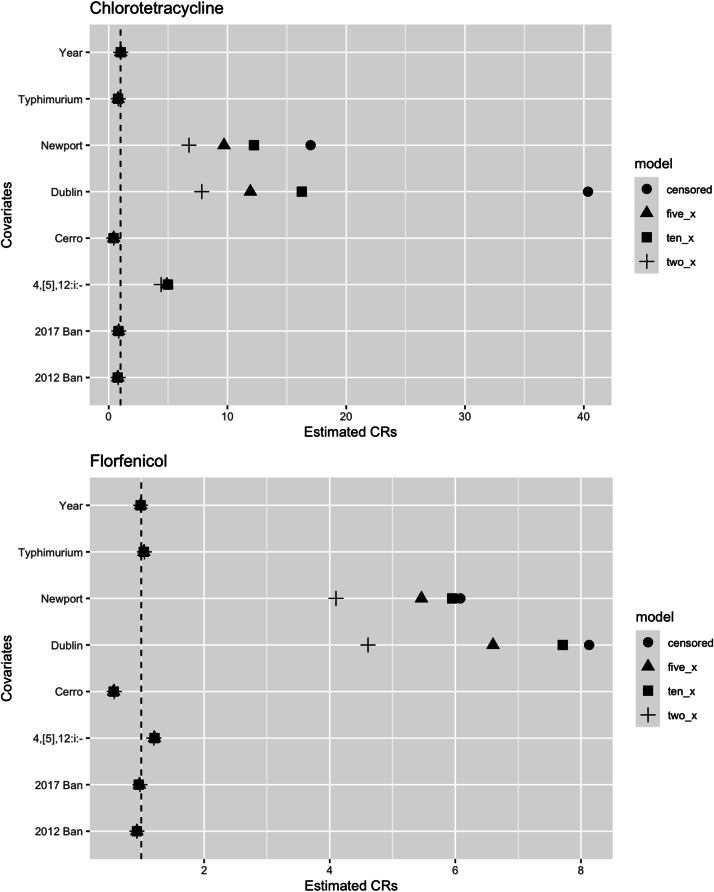

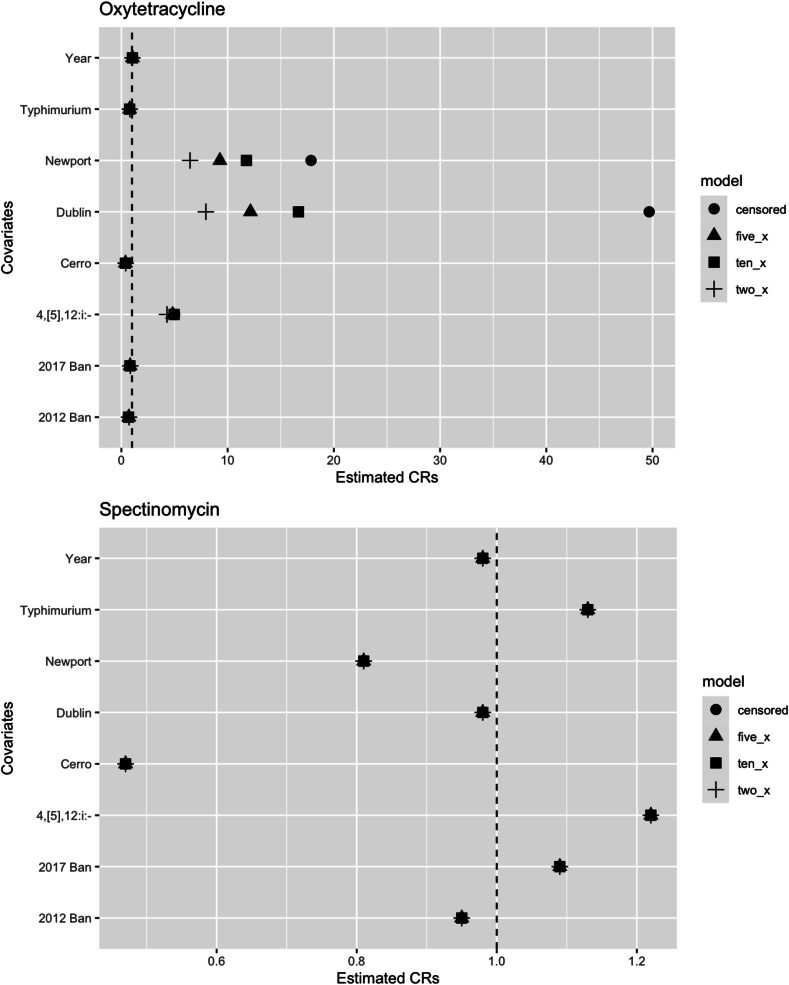
A comparison of the estimated concentration ratios (CRs) for each covariate in an AFT model with censored data, censored data replaced with concentrations 2× the highest concentration tested, censored data replaced with concentrations 5× the highest concentration tested, or censored data replaced with concentrations 10× the highest concentration tested. The dashed line indicates a null CR value of 1.

A sensitivity analysis was done to evaluate the impact of censored data on the estimated concentration ratios (CR) of the AFT model. Censored right limits were replaced with values two, five, or ten times the highest concentration tested against a given isolate. After re-fitting the AFT models, we found that the statistical significance and direction of the CR were unchanged for all covariates in all AFT models. For each 2×, 5×, and 10× AFT model that was assessed, the CRs for the 2012 and 2017 restriction differed by less than 12 % compared to the model that included the censored data. The CRs differed by less than 17 % for serotypes 4, [5],12:i:-, Cerro, and Typhimurium in comparison to the model that included censored data. *Salmonella* Dublin and Newport demonstrated the most variation in MIC values across the different model types (Fig. 2).

## 4 Discussion

This study investigated the effect of AMU regulations on the AMR and MIC trends among *Salmonella* isolates from bovine samples submitted to a veterinary diagnostic laboratory. Logistic regression models were used to examine proportional changes in the prevalence of resistance in response to the adoption of AMU restrictions in dairy cattle populations, whereas AFT models were used to evaluate changes in MIC values associated with the same AMU restrictions.

Interestingly, the 2012 AMU restriction on the extra-label use of cephalosporins in food producing animals was found to be significantly associated with a decrease in resistance to chlortetracycline, oxytetracycline, and neomycin but had no statistically significant relationship with ceftiofur resistance or resistance to other antimicrobials when controlling for serotype and year of isolation. The 2012 restriction on extra-label use of cephalosporins still allows use of these drugs to treat or control extra-label disease indications, as long as this use is in accordance with the labeled dose, route, and duration. This effectively can be viewed as a restriction on quantity of drug administered to a given animal, which might not be sufficient to have an appreciable impact on resistance to ceftiofur. Studies aimed at quantifying AMU on Pennsylvania and Wisconsin dairy farms noted first- and third-generation cephalosporins to be among the most frequently used drug classes [28,29]. Similarly, New York dairy farmers mentioned cephalosporins as a frequent choice of treatment for diseases such as mastitis, metritis, and respiratory disease [30]. AMU rates were found to be lower across the study period in all drug classes in comparison to AMU in other states, except for cephalosporins in Pennsylvania [29]. It is possible that a similar trend of frequent cephalosporin use can be observed in many U.S. states, thus promoting an increase in the prevalence of plasmids carrying β-lactam resistance genes and consequently other AMR genes. Resistance to cephalosporins is frequently conferred by β-lactamase genes such as *blaCMY* and *blaTEM* [31]. AMR genes conferring resistance to β-lactams have been detected on the same plasmids as genes conferring resistance to aminoglycosides, folate pathway inhibitors, and tetracyclines [32]. Plasmids may be maintained by selection pressure from any single antimicrobial drug for which there is resistance. There is a need for the quantification of AMU in more U.S. states to understand the connection between AMU and AMR in agricultural settings.

The decrease in the proportion of *Salmonella* isolates resistant to chlortetracycline, oxytetracycline, and neomycin in association with the 2012 AMU restriction is an encouraging observation. Corresponding decreases in MIC values were observed for chlortetracycline and oxytetracycline. Nobrega et al. similarly noted that a decrease in the prevalence of genes conferring resistance to tetracyclines in bacterial pathogens isolated from cattle, swine, and poultry samples was associated with AMU interventions [33].

Except for florfenicol, the 2017 restriction on in-feed AMU in food-producing animals was not significantly associated with changes in the proportion of resistant isolates or changes in MIC values. According to a USDA National Animal Health Monitoring System (NAHMS) report, medicated milk replacers (primarily including neomycin and tetracycline drugs, alone or in combination) were the primary use of antimicrobials for growth promotion purposes in U.S. dairy cattle prior to the 2017 restriction [34]. Therefore, we expected a decrease in resistance to neomycin and tetracyclines after 2017, yet this was not the case. A plausible explanation for the lack of association between resistance to oxytetracycline, chlortetracycline, and neomycin and the 2017 restriction in this study is the relative decrease in sample size after 2017. Sakar and Okafor et al. identified a significant increase in the proportion of tetracycline-resistant *Salmonella* isolated from retail meats in association with the 2017 AMU restriction [21]. Sakar and Okafor used a larger sample size and used pre-2017 as the reference category while our analysis used pre-2012 as the reference category in the statistical models, which may explain the difference in results [21]. The authors further noted that the observed increase in resistance may have been a result of serotype trends [21]. According to the FDA, tetracyclines represent the largest volume of antimicrobial sales and are considered medically important; thus, preserving their effectiveness and reducing resistance is essential [16].

It is unclear why there was an observed association between florfenicol resistance and the 2017 AMU restriction. There are several factors that contribute to the development of AMR but were not included in our statistical models due to data limitations. For example, herd management practices, biosecurity measures, disease incidence, and serotype trends can influence AMR. The association with florfenicol resistance could be further explored through the assessment of comprehensive and high-quality data on U.S. dairy herds.

Serotype was an important predictor of AMR and MIC trends. Our previous study showed serotype to be a significant driver of AMR trends [26]. A study investigating AMU and AMR in Canadian poultry farms similarly concluded *Salmonella* serotype has an important impact when interpreting AMR trends [35]. Our chi-squared tests of association demonstrated a significant statistical association between AMU restrictions and AMR for most of the antimicrobials. However, when controlling for serotype and year of isolation, the AMU restriction had no effect on seven out of 10 of the antimicrobials assessed by logistic regression or AFT modeling. Our results suggest that the impact of AMU restrictions varies across antimicrobials and that serotype-specific targeted interventions alongside improved detection methods may be required to reduce or eliminate highly resistant *Salmonella* serotypes and increase the effectiveness of AMU restrictions.

Because the AFT models include censored observations, it is important to assess the impact of censoring on CR interpretations. The sensitivity analysis revealed Dublin and Newport to have high variation in CR. Particularly, the AFT models that replaced censored values with an MIC value 2× the highest concentration tested displayed the greatest difference in estimated CRs. The AFT models that replaced censored values with an MIC value 10× the highest concentration tested displayed the smallest difference compared to the censored model. This observation was expected because Dublin and Newport exhibit the highest proportion of resistance and thus have high proportions of right-limit censored observations [26]. In each AFT model (censored, 2×, 5×, 10×), the model predicted relatively higher MIC values for *Salmonella* Dublin and Newport isolates, which aligns with the overall AMR profile for these two serotypes [36,37]. The relatively smaller variation in estimated CRs between the censored, 2×, 5×, and 10× models for 4, [5],12:i-, Cerro, and Typhimurium supports the notion that these isolates do not generally have high MIC values and are not sensitive to changes in MIC values in other serotype categories. The consistency in *p*-values and directions of significant relationships suggest that AFT models are appropriate for assessing changes in MIC values when there is sufficient variability in these values.

The logistic regression models and the corresponding AFT models revealed similar conclusions; more than 50 % of the covariates in the models overlapped in the direction and significance of the covariates. The antimicrobial models that had a significant covariate in the logistic regression model but not the corresponding AFT model (ceftiofur, florfenicol, and spectinomycin) suggest that isolates belonging to a given covariate category have significant variability in the proportion of isolates that are resistant; however, all the resistant isolates had similar MIC values, and all the susceptible isolates had similar MIC values. For example, the spectinomycin AFT model indicated the year and serotype 4, [5],12:i- to be significant predictors of resistance, whereas the corresponding logistic regression did not. The spectinomycin AFT model appears to be more sensitive to changes across years, which may explain the discordance between models.

There are inherent biases in working with diagnostic laboratory data. For example, the results presented here may not represent subclinically infected animals. Additionally, we do not have information on the genomic profiles of the isolates tested or characteristics of the farms from which the animals originated. Therefore, we are unable to statistically associate AMR genes, virulence factors, or farm characteristics with the observed AMR and MIC trends. Because our study focused on *Salmonella* isolated from bovine samples, these results may not apply to other bacterial and animal species. For example, Sarkar and Okafor et al. examined the impact of the Veterinary Feed Directive on AMR in retail meats and noted the odds of detecting tetracycline-resistant *Salmonella*, *Campylobacter*, or *Escherichia coli* in retail meats varied across bacteria and meat type [21]. Lastly, we must emphasize that the statistical associations shown here do not imply causal relationships; additional AMU data and analysis would be needed to make such inferences. Currently, AMU data are not publicly available to effectively link AMR trends and AMU reductions.

## 5 Conclusions

Our study, based on veterinary diagnostic laboratory data on *Salmonella* isolates from cattle over a 16-year period, has shown that when controlling for serotype and year of isolation, AMU restrictions were associated with either no effect or a positive effect (decrease in odds of resistance or decrease in MIC values) for the antimicrobials assessed via logistic regression or AFT models. Because serotype is an important indicator of resistance to antimicrobials, it is important to consider serotype-specific biosecurity measures, farm hygiene practices, and antimicrobial treatment protocols to reduce the presence of *Salmonella* and associated AMR genes. Our study helps to strengthen knowledge of how federal policies regarding livestock AMU influence AMR and MIC patterns of *Salmonella* isolated from bovine samples.

## CRediT authorship contribution statement

**Maya Craig:** Writing – review & editing, Writing – original draft, Visualization, Formal analysis. **Kevin J. Cummings:** Writing – review & editing, Supervision, Funding acquisition, Conceptualization. **Claudia Cobo-Angel:** Writing – review & editing, Formal analysis. **Casey L. Cazer:** Writing – review & editing, Supervision, Funding acquisition. **Melissa S. Aprea:** Writing – review & editing, Data curation. **Rebecca J. Franklin-Guild:** Writing – review & editing, Data curation.

## Funding

This work is supported by a Hatch award under 7,000,403 from the USDA National Institute of Food and Agriculture.

## Declaration of competing interest

The authors declare no conflicts of interests.

## Data Availability

Data will be made available on request.

## Appendix A Supplementary data

Supplementary material Image 1

## References

[1] Llor C., Bjerrum L. (2014). Antimicrobial resistance: risk associated with antibiotic overuse and initiatives to reduce the problem. Therap. Adv. Drug Saf..

[2] Marshall B.M., Levy S.B. (2011). Food animals and antimicrobials: impacts on human health. Clin. Microbiol. Rev..

[3] Manyi-Loh C., Mamphweli S., Meyer E., Okoh A. (2018). Antibiotic use in agriculture and its consequential resistance in environmental sources: potential public health implications. Molecules.

[4] Tang K.L., Caffrey N.P., Nóbrega D.B., Cork S.C., Ronksley P.E., Barkema H.W., Polachek A.J., Ganshorn H., Sharma N., Kellner J.D., Ghali W.A. (2017). Restricting the use of antibiotics in food-producing animals and its associations with antibiotic resistance in food-producing animals and human beings: A systematic review and meta-analysis. Lancet Planet. Health.

[5] Hu Y., Yang X., Li J., Lv N., Liu F., Wu J., Lin I.Y.C., Wu N., Weimer B.C., Gao G.F., Liu Y., Zhu B. (2016). The bacterial mobile resistome transfer network connecting the animal and human microbiomes. Appl. Environ. Microbiol..

[6] Xu C., Kong L., Gao H., Cheng X., Wang X. (2022). A review of current bacterial resistance to antibiotics in Food animals. Front. Microbiol..

[7] Holschbach C.L., Peek S.F. (2008). *Salmonella* in dairy cattle. Vet. Clin. North Am. Food Anim. Pract..

[8] World Health Organization (2018). *Salmonella* (Non-Typhoidal). https://www.who.int/news-room/fact-sheets/detail/salmonella-(non-typhoidal).

[9] Patel, K., Stapleton, G. S., Trevejo, R. T., Tellier, W. T., Higa, J., Adams, J. K., Hernandez, Sonia M., Sanchez, S., Nemeth, N.M., Debess, E.E., Rogers, K.H., Mete, A., Watson, K.D., Foss, L., Low, M.S.F, Gollarza, L., Nichols, M. (2023) Human salmonellosis outbreak linked to *Salmonella* typhimurium epidemic in wild songbirds, United States, 2020–2021. Emerg. Infect. Dis. J. 29*,* 11. doi: 10.3201/eid2911.230332.PMC10617330 37877570

[10] Gutema F.D., Agga G.E., Abdi R.D., De Zutter L., Duchateau L., Gabriël S. (2019). Prevalence and serotype diversity of *Salmonella* in apparently healthy cattle: systematic review and meta-analysis of published studies, 2000–2017. Front. Vet. Sci..

[11] An K., Wu Z., Zhong C., Li S. (2023). Case report: uncommon presentation of *Salmonella* Dublin infection as a large paravertebral abscess. Front. Med..

[12] Ford L. (2023). Strain of multidrug-resistant *Salmonella* Newport remains linked to travel to Mexico and U.S. beef products—United States, 2021–2022. MMWR Morb. Mortal Wkly. Rep..

[13] Burciaga S., Trachsel J.M., Sockett D., Aulik N., Monson M.S., Anderson C.L., Bearson S.M.D. (2023). Genomic and phenotypic comparison of two variants of multidrug-resistant *Salmonella enterica* serovar Heidelberg isolated during the 2015–2017 multi-state outbreak in cattle. Front. Microbiol..

[14] Marshall K.E.H. (2018). Protracted outbreak of *Salmonella* Newport infections linked to ground beef: possible role of dairy cows — 21 states, 2016–2017. MMWR Morb. Mortal Wkly. Rep..

[15] U.S. Food and Drug Administration (2012). https://www.govinfo.gov/content/pkg/FR-2012-01-06/pdf/2012-35.pdf.

[16] Food U.S., Administration Drug (2022). 2021 Summary Report On Antimicrobials Sold or Distributed for Use in Food-Producing Animals. https://www.fda.gov/animal-veterinary/cvmupdates/fda-releases-annual-summary-report-antimicrobials-sold-or-distributed-2021-use-foodproducing.

[17] World Health Organization (2019). WHO List of Medically Important Antimicrobials. https://cdn.who.int/media/docs/default-source/gcp/who-mia-list-2024-lv.pdf?sfvrsn=3320dd3d_2.

[18] Nair V.T., Venkitanarayanan K., Kollanoor Johny A. (2018). Antibiotic-resistant *Salmonella* in the Food supply and the potential role of antibiotic alternatives for control. Foods.

[19] U.S. Food and Drug Administration (2024). https://www.fda.gov/animal-veterinary/development-approvalprocess/fact-sheet-veterinary-feed-directive-final-rule-and-next-steps.

[20] Zawack K., Li M., Booth J.G., Love W., Lanzas C., Gröhn Y.T. (2016). Monitoring antimicrobial resistance in the food supply chain and its implications for FDA policy initiatives. Antimicrob. Agents Chemother..

[21] Sarkar S., Okafor C. (2023). Effect of veterinary feed directive rule changes on tetracycline-resistant and erythromycin-resistant bacteria (*Salmonella, Escherichia*, and *Campylobacter*) in retail meats in the United States. PLoS ONE.

[22] Fedorov V., Mannino F., Zhang R. (2009). Consequences of dichotomization. Pharm. Stat..

[23] Irwin J. (2003).

[24] Cobo-Angel C., Mosaddegh A., Aprea M., Guarino C., Cummings K.J., Cazer C. (2023). Trends of feline *Escherichia* coli minimum inhibitory concentrations over 14 years illustrate the need for judicious antimicrobial use in cats. Am. J. Vet. Res..

[25] Osman M., Altier C., Cazer C. (2023). Antimicrobial resistance among canine enterococci in the northeastern United States, 2007–2020. Front. Microbiol..

[26] Craig M.J., Cummings K.J., Aprea M.S., Franklin-Guild R.J., Altier C. (2024). Serotype and anti-microbial resistance trends among bovine *Salmonella* isolates from samples submitted to a veterinary diagnostic laboratory in Central New York, 2007–2021. Zoonoses Public Health.

[27] Center for Disease Control and Preventation (CDC) (2019). Antibiotics Tested by NARMS | NARMS | CDC. https://www.cdc.gov/narms/antibiotics-tested.html.

[28] Campos J.L., Kates A., Steinberger A., Sethi A., Suen G., Shutske J., Safdar N., Goldberg T., Ruegg P.L. (2021). Quantification of antimicrobial usage in adult cows and preweaned calves on 40 large Wisconsin dairy farms using dose-based and mass-based metrics. J. Dairy Sci..

[29] Redding L.E., Bender J., Baker L. (2019). Quantification of antibiotic use on dairy farms in Pennsylvania. J. Dairy Sci..

[30] Ekakoro J.E., Caldwell M., Strand E.B., Okafor C.C. (2018). Drivers of antimicrobial use practices among Tennessee dairy cattle producers. Vet. Med. Int..

[31] Carattoli A. (2008). Animal reservoirs for extended spectrum β-lactamase producers. Clin. Microbiol. Infect..

[32] Michael G.B., Butaye P., Cloeckaert A., Schwarz S. (2006). Genes and mutations conferring antimicrobial resistance in *Salmonella*: an update. Microbes Infect..

[33] Nobrega D.B., Tang K.L., Caffrey N.P., De Buck J., Cork S.C., Ronksley P.E., Polachek A.J., Ganshorn H., Sharma N., Kastelic J.P., Kellner J.D., Ghali W.A., Barkema H.W. (2021). Prevalence of antimicrobial resistance genes and its association with restricted antimicrobial use in food-producing animals: a systematic review and meta-analysis. J. Antimicrob. Chemother..

[34] National Animal Health Monitoring System (NAHMS) (2007). https://www.aphis.usda.gov/sites/default/files/dairy07_is_antibioticuse.pdf.

[35] Huber L., Agunos A., Gow S.P., Carson C.A., Van Boeckel T.P. (2021). Reduction in antimicrobial use and resistance to *Salmonella, Campylobacter*, and *Escherichia coli* in broiler chickens, Canada, 2013–2019. Emerg. Infect. Dis..

[36] Srednik M.E., Lantz K., Hicks J.A., Morningstar-Shaw B.R., Mackie T.A., Schlater L.K. (2021). Antimicrobial resistance and genomic characterization of *Salmonella* Dublin isolates in cattle from the United States. PLoS ONE.

[37] Carroll L.M., Wiedmann M., den Bakker H., Siler J., Warchocki S., Kent D., Lyalina S., Davis M., Sischo W., Besser T., Warnick L.D., Pereira R.V. (2017). Whole-genome sequencing of drug-resistant *Salmonella enterica* isolates from dairy cattle and humans in New York and Washington states reveals source and geographic associations. Appl. Environ. Microbiol..

