# Supplementary material for: The impact of antimicrobial use regulations on antimicrobial resistance among Salmonella isolates from bovine samples submitted to a veterinary diagnostic laboratory in Central New York
Source: One Health. 2025 Jun 2;20:101087. doi: 10.1016/j.onehlt.2025.101087 (PMC12179662; doi:10.1016/j.onehlt.2025.101087)
Supplement: Supplementary file 1 — Supplementary material [file mmc1.docx]

**SUPPLEMENTARY MATERIALS**

**Table 1**. Proportion of *Salmonella* Dublin isolated from bovine samples that are resistant to **Sulfadimethoxine**

|  | **Resistance** | **Susceptible** |
| --- | --- | --- |
| **Dublin** | 276 | 0 |

**Table 2.** Distribution of *Salmonella* Newport ceftiofur MIC values

|  | **≤ 0.25** | **0.5** | **1** | **2** | **4** | **>4** | **8** | **>8** | **Total** |
| --- | --- | --- | --- | --- | --- | --- | --- | --- | --- |
| TIO | 0 | 3 | 9 | 0 | 4 | 1 | 0 | 137 | 154 |
